# On the Effects of the Jacca, or Viola Tricolor, Linn. in Curing Venereal Ulcers, and Other Venereal Remains

**Published:** 1805-11-01

**Authors:** 


### On the Effects of the Jacca, or Viola Tricolor, Linn. in curing Venereal Ulcers, and other Venereal Remains.

Dr. SCHLEGEL, of Moscow, communicated last year his observations on the jacca officinalis, for the cure of veneral ulcers. The result of which must claim the attention tion of every professional man. However, we cannot forbear laying before our readers some cases, where the boasted effect of the jacca proved very doubtful, at least, as a radical cure.

A countryman, by profession a weaver, had for several years a venereal eruption at his nose, lips and chin, which resisted every medicine he hitherto had taken. On examination, the velum palati was likewise found ulcerated, and besides, some blotches appeared on different parts of the body. According to Dr. Schlegel, half an ounce of the herba jacca was ordered to be boiled in two pounds of water, until reduced to half the quantity, of which half a tea cup full was taken every two hours. The same decoction was applied outwardly, for gargling, and for constantly moistening the eruption. Three days after, the whole had a better appearance. After six days the crusts dryed off, and by the continued use of it four weeks, the face was entirely free from eruptions. The patient finding himself so much relieved, constantly took the decoction of the jacca, which was gradually increased in strength. At the end of the sixth week, an inflammation was spreading over all the infected parts, and as this was thought owing to the jacca, it was left off, when the inflammation subsided. Three weeks after the ulcer in the throat was much increased, and the places where the eruption occurred before, began to itch, and became painful and burning. The jacca was again ordered in smaller quantity, but the inflammation rose to such a degree, that it was thought proper to leave it off entirely, and finish the cure with mercurials, &c.

A girl, aged twenty, was infected with an ulcer in her throat during three-quarters of a year. The decoction of the jacca was prescribed, for gargling, and inward use. The first days she found much relief, but soon after the inflammation was considerably increased, and she suffered a great deal of pain. The quantity of the jacca was diminished, in consequence of which the inflammation subsided, but the ulceration gained more ground; after a continued use of the jacca for four weeks, it became necessary to leave it off, and to apply mercurials, by which the cure was speedily effected.

